# Comparative Evaluation of Two Different Post-Operative Analgesia after Hallux Valgus Correction in Day Surgery Patients

**DOI:** 10.5704/MOJ.2007.013

**Published:** 2020-07

**Authors:** M Galli, A Vergari, R Vitiello, R Nestorini, M Peruzzi, A Chierichini, G Spinazzola, M Rossi

**Affiliations:** 1 Department of Orthopaedics and Traumatology, Universita Cattolica del Sacro Cuore, Rome, Italy; 2 Department of Anaesthesiology, Universita Cattolica del Sacro Cuore, Rome, Italy; 3 Department of Orthopaedics and Traumatology, IRCCS Fondazione Policlinico Universitario Agostino Gemelli, Rome, Italy

**Keywords:** hallux valgus, SCARF osteotomy, regional anaesthesia, pain, ERAS

## Abstract

**Introduction:**

The aim of this study was the evaluation of two different techniques on post-operative analgesia and motor recovery after hallux valgus correction in one-day surgery patients.

**Material and Methods:**

We enrolled 26 patients scheduled for hallux valgus surgery and treated with the same surgical technique (SCARF osteotomy). After subgluteal sciatic nerve block with a short acting local anaesthetic (Mepivacaine 1.5%, 15ml), each patient received an ultrasound-guided Posterior Tibialis Nerve Block (PTNB) with Levobupivacaine 0.5% (7-8ml). We measured the postoperative intensity of pain using a Visual Analogue Scale (VAS), the consumption of oxycodone after operative treatment and the motor recovery. VAS was detected at baseline (time 0, before the surgery) and at 3, 6, 12 and 24 hours after the operative procedure (T1, T2, T3, T4 respectively). Control group of 26 patients were treated with another post-operative analgesia technique: local infiltration (Local Infiltration Anaesthesia, LIA) with Levobupivacaine 0.5% (15ml) performed by the surgeon.

**Results:**

PTNB group showed a significant reduction of VAS score from the sixth hour after surgery compared to LIA group (p<0.028 at T2, p<0.05 at T3 and p<0.002 at T4, respectively). Instead, no significant differences were found in terms of post-operative oxycodone consumption and motor recovery after surgery.

**Conclusions:** PTNB resulted in a valid alternative to LIA approach for post-operative pain control due to its better control of post-operative pain along the first 24 hours. In a multimodal pain management according to ERAS protocol, both PTNB and LIA should be considered as clinically effective analgesic techniques.

## Introduction

Hallux valgus correction is often performed as an inpatient procedure due to concerns regarding adequate post-operative analgesia. Outpatient surgery, also known as ambulatory surgery, same-day surgery, day case, or day surgery, is a surgery that does not require an overnight hospital stay. Ideal anaesthesia for outpatient surgery procedures should provide rapid patient recovery, minimal nursing care requirements in the post-anaesthesia care unit and earlier hospital discharge. It must provide effective analgesia, since foot surgery is known to induce a moderate to severe post-operative pain that represents one of the major limitations to hospital discharge on the day of surgery and is the main cause of readmission^1, 2^.

Multimodal analgesia is one of the pillars of Enhanced Recovery After Surgery (ERAS) protocols and relies on opioid-sparing and combined actions of drugs and antalgic techniques^3, 4^. In hallux valgus surgery sciatic nerve block with long acting local anaesthetics (with or without a perineural catheter) has been advocated as a primary option^5-13^, to avoid the administration of large doses of parenteral opioids due to significant post-operative pain^5-8^. This analgesic approach may cause a difficult discharge in ambulatory and one-day surgery patients, due to the prolonged motor recovery and loss of proprioception and protective pain reflexes^14, 15^. Sciatic nerve block (just for intra-operative anaesthesia without affecting motor recovery) and multimodal analgesia for post-operative pain management, instead, may accelerate patient recovery and discharge.

Most of the traditional hallux valgus and forefoot surgical techniques has already met the ERAS requirements, they allow immediate mobilisation and weight bearing, but early patient recovery is significantly hampered by post-operative pain. Multimodal analgesia may contribute to the achievement of an adequate post-operative pain control along with rapid patient discharge.

This matched control study aimed to evaluate two different techniques of multimodal post-operative analgesia after hallux valgus correction in one-day surgery patients.

## Materials and Methods

**Fig. 1:**
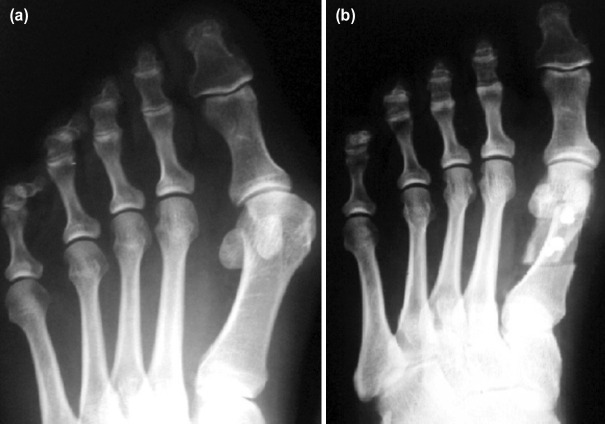
(a) The pre-surgery radiograph. (b) The post-surgery radiograph after performing a SCARF osteotomy.

The study was performed in our Hospital and was approved by the Hospital Ethics Committee (ID: 50990/17), Clinical Trial registry number NCT03396991. Patient consent was obtained according to the Italian Regulations. The study was designed following TREND guidelines. During the study period (1 July 2017 – 1 October 2017), twenty-six consecutive ASA physical status I or II patients (study group, SG) scheduled for elective hallux valgus surgery were enrolled. All patients had a standard radiographic examination before surgery and unilateral procedures. The inclusion criteria were: moderate symptomatic hallux valgus (according to Coughlin^16^ radiographic classification) undergoing surgical correction by a SCARF procedure (Table I; Fig. 1). All the patients present an intermetatarsal angle <20° and hallux valgus angle <40°. SCARF procedure was performed according to the description popularised by Barouk^17^ in the year 2000 and fixation was achieved by the insertion two 3.0 cannulated Herbert compression screws. The mean time of surgery was 60±15 min, no statistical difference between SG and CG group. Whenever an adjunctive first ray proximal phalanx osteotomy was needed to complete the surgical correction of the deformity, the data was recorded and the patient was not discarded (Table I). Proximal phalanx osteotomies were performed according to Akin as described by Frey18 and fixed using staples. All patients were operated on by the same surgeon: our senior orthopaedic surgeon for foot and ankle diseases. Patients who underwent adjunctive lesser toes and/or lesser metatarsal surgery and patients with peripheral circulatory disorders, skin lesions of the foot or allergy to local anaesthetic were excluded as well as patients routinely assuming analgesics or history of analgesic consumption within 24 hours before surgery.

**Table I:** Patients characteristics

	SG (N = 26)	CG (N = 26)	P
Age (yr)	59.38 ± 14.45	58.58 ± 13.52	ND
BMI (Kg/m^2^)	24.46 ± 2.95	2.,09 ± 5.04	ND
ASA	ASA 1	ASA 1	
	N = 8 (31%)	N = 8 (31%)	ND
	ASA 2	ASA 2	
	N = 18 (69 %)	N = 18 (69 %)	
Gender	Male	Male	
	N = 4 (15%)	N = 4 (15%)	ND
	Female	Female	
	N = 22 (85%)	N = 22 (85 %)	
Adjunctive Akin Procedures	N = 8	N = 6	
	Freq. = 30:8%	Freq. = 23.1%	ND

Data are presented as mean ± standard deviation, ND: no statistical differences, NP: not presented. Study Group (SG), Control Group (CG), American Society Anaesthesiologists Physical Status Classification System (ASA).

Before the operative procedure, all patients received a subgluteal sciatic nerve block performed by an experienced practitioner. After having positioned the patient in the lateral decubitus position, the subgluteal sciatic nerve block was realised with a short bevel, insulated needle (70mm long, 22gauge, Temena, Germany), under ultrasound control plus nerve stimulator. Once the nerve was identified the needle was inserted in plane and after careful aspiration to rule out an intravascular needle placement a short acting local anaesthetic (Mepivacaine 1.5%, 15ml) was injected.

**Fig. 2:**
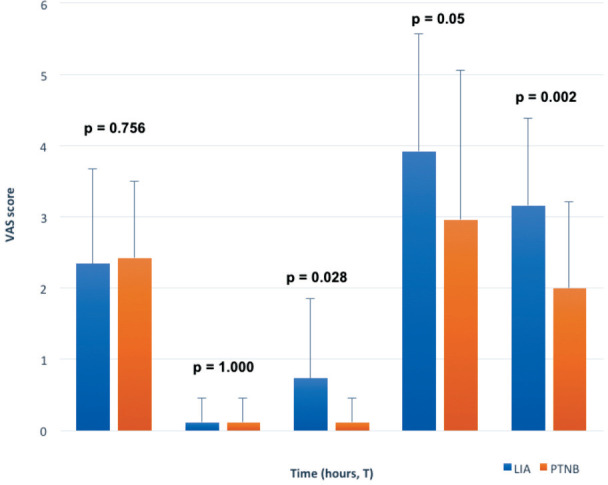
Ultrasound guided posterior tibialis nerve block. (LIA: Local Infiltration Anaesthesia; PTNB: Posterior Tibialis Nerve Block).

After sciatic nerve block, each patient of the SG received an ultrasound guided Posterior Tibialis Nerve Block (PTNB). The PTNB was performed with the patient in supine position and the foot externally rotated. The short bevel insulated needle (35mm long, Temena, Germany), was inserted, with ultrasound control, in plane and posteriorly to the tibial artery and 7-8ml of a long acting anaesthetic (Levobupivacaine 0.5%) was injected (Fig. 2).

After the operative procedure, the patients were monitored for one hour in the Recovery Room and then transferred to the ward. All the patients received Ketoprofen (50mg) and Paracetamol (1000mg) orally every eight hours, starting four hours after the end of procedure until discharge from the hospital. Oxycodone 5mg, maximum 20mg/24h, was given if the pain intensity was more than 5. After discharge from the hospital, the patients continued analgesic therapy for another three days with oral Paracetamol 1000mg three times a day and oral Ketoprofen 50mg twice daily.

The SG was matched with a control group (CG) of twenty-six patients, scheduled for the same surgery, same demographic data, in a chosen control methodology. The CG group was treated with the same anaesthetic technique (sciatic nerve block) but with a different post-operative analgesia technique: an infiltration (Local Infiltration Anaesthesia, LIA) of long acting local anaesthetic (Levobupivacaine 0.5%, 15ml) performed by the surgeon directly on the operative site after an initial negative aspiration test, using a 21 gauge short bevel needle (40mm long, BD microline, Milan, Italy). The CG received the same post-operative pain management with oral Ketoprofen, Paracetamol, and Oxycodone as needed. The primary endpoint of the study was the pain control measured by a visual analogue scale of Scott-Huskisson (VAS, 0 lack of pain, 10 the worst pain). VAS was detected at baseline (time 0, before the surgery) and at 3, 6, 12 and 24 hours after the operative procedure (T1, T2, T3, T4 respectively). The secondary endpoints were the Oxycodone consumption in the first 24 hours after operative treatment and the duration of motor block measured by the Modified Bromage Score (I Free movement of legs and feet, II Just able to flex knees with free movement of feet, III Unable to flex knees, but with free movement of feet, IV Unable to move legs or feet).

All data are expressed as mean ± standard deviation (DS). The Kolmogorov-Smirnov test was used to determine the data normal distribution. We applied the “Statistical Package for Social Science program (SPSS)” version 15.0 for statistical analysis. For the calculation of the sample size, data from a pilot study on 10 subjects were used. A significant difference of the VAS score in the first 24 hours of intervention was defined as the primary endpoint.

In the pilot study, the mean post-operative VAS was 4. Estimating a decrease of at least 10% of the VAS as significant, it necessary to recruit 12 patients per group. This number was increased of 40% to reduce any bias due to non-randomisation (α = 0.05, β = 80%). Patients in the two groups were matched based on demographic and surgical variables. After matching, the two groups appeared homogeneous.

We used the two-tailed t test to compare the VAS values between the two different analgesia techniques. Because the duration of motor block and oxycodone consumption did not follow a normal distribution, Mann-Whitney U tests were used to compare these two outcomes. P values ≤ 0.05 were considered statistically significant.

## Results

**Fig. 3:**
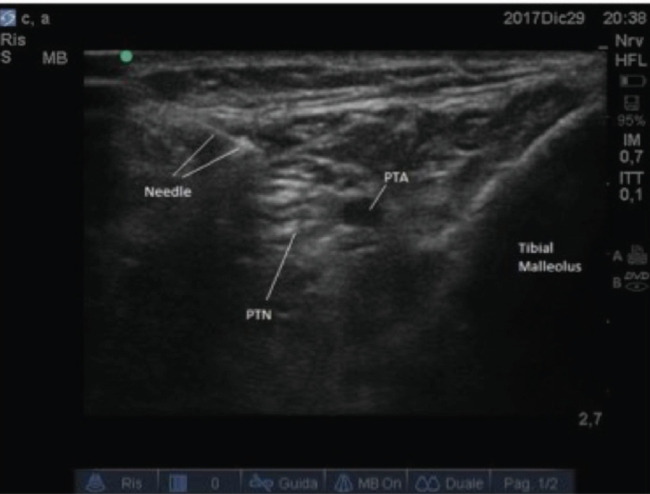
Variation of pain in the first day after surgery. At T0 and T1 no significant differences could be detected in the VAS score but from T2 until discharge from the hospital the study group presented a significant reduction of VAS. T0: before the surgery; T1 3 hours after surgery; T2 6 hours after 318 surgery; T3 12 hours after surgery; T4 24 hours after surgery. (PTN: posterior tibialis nerve; PTA: posterior tibialis artery).

Twenty-six patients per group were recruited; the two groups were found to be homogeneous in demographic and surgical variables (Table I). All patients underwent surgery without requiring sedation or supplemental analgesia. During the first three hours no significant differences could be detected in terms of VAS values between the two analgesic techniques, but from the sixth hour after surgery until discharge from the hospital the SG presented a significant reduction of VAS values compared to CG (p<0.028 at T2, p< 0.05 at T3 and p< 0.002 at T4, respectively) (Fig. 3).

In terms of post-operative oxycodone consumption, no significant differences could be detected between groups. 19 patients (73%) in the SG group and 20 patients (78%) in the CG (p>0.89) needed the opioid during the first 24 postoperative hours. The first oxycodone administration occurred in both groups eight hours after the operative procedure at the least and the average dose administered in both groups was 7mg (6.73 ± 5.28 in SG, 7.31 ± 5.14 in CG, p = 0.69). The duration of motor block, evaluated by Bromage Score, was 3 ± 0.5 hours in each group and no significant differences between the two techniques were found.

Starting from the third hour after surgery, all patients were able to stand and walk with the aid of canes, partial weight bearing and a post-operative shoe (Podalux DonJoy®). No side effects were recorded during all post-operative hospital stay in both groups. All patients were discharged from the hospital on the first post-operative day with the recovery of free walking capacity and full weight bearing with postoperative bandage and shoe. Canes were nevertheless recommended together with rest and foot elevation to prevent excessive local swelling in the first post-operative week. At one-month follow-up neither significant complications nor cases of chronicization of post-operative pain were recorded.

## Discussion

Although hallux valgus surgery is included into the twenty most painful surgeries, and the relationship between a poorly controlled post-operative pain and its chronicity is well known^19, 20^, there is an increasing demand from patients and health care systems for foot surgery to be performed in an ambulatory or day surgery setting.

Regional anaesthesia techniques commonly used for hallux valgus surgery such as sciatic nerve block and ankle blocks have not been studied about their benefit on post-operative pain, on the contrary, their possible negative effect on a rapid motor recovery is well known^21^. The safety of discharging patients with a long-acting sciatic block remains controversial^14^. Because of the loss of proprioception and protective pain reflexes, outpatients are at risk of falls, trauma, inability to ambulate, and accidental injury of the limb at the surgical site^15^.

Ideal anaesthesia should provide rapid self-sufficient ambulation recovery for a fast hospital discharge and should minimise nursing care. Although a rapid mobilisation might be achieved with a selective and short-acting anaesthetic sciatic block the problem of an adequate post-operative pain control remains.

ERAS protocols have been recently implemented in orthopaedic surgery, and the two cornerstones are early mobilisation and multimodal analgesia^22-24^. Following the ERAS principles, our study has focused on post-operative analgesic effects of two different analgesic techniques after hallux surgery, associated with a selective and short-acting anaesthetic sciatic block, and a multimodal pharmacological approach. The first analgesic technique takes its move from already validated anaesthetic/analgesic techniques for Hallux surgery such as popliteal sciatic nerve block^14^. In our case, a more distal nerve block was adopted (PTNB) to spare the motor innervation of calf muscles.

The other is a multimodal, opioid-sparing regimen, which has already been advocated for its effectiveness in Total Knee Arthroplasties and consists of a local infiltration analgesia (LIA) with anaesthetics that cover the initial 6 to 12 post-operative hours^25^. The recent American guidelines for the management of post-operative pain^26^ and the ERAS statements^3, 4^ recommend the search of less invasive approaches, preserving the combination of pharmacological and non-pharmacological modalities. LIA presents the disadvantage of infiltrating high volumes of long acting anaesthetic and is less reproducible, but it spares a second nerve block. PTNB instead spares high volumes of anaesthetic and is more reproducible, but consists of a second sciatic nerve block, with the theoretical risk of local anaesthetic intravascular injection or mechanical nerve injury.

The main finding of our study was the significant analgesic efficacy of PTNB compared to LIA during the first postoperative period after hallux valgus surgery. Although the study allowed us to meet our primary endpoint, with the sample dimension derived from the least clinical difference in pain perception as measured by VAS no significant differences could be detected in terms of analgesic requirements and motor recovery between the two groups. In any case, in our view, the present outcome is sufficient to point out that PTNB is superior to LIA in handling the postoperative pain along the 24 hour after surgery. However, both techniques are substantially comparable in resisting to pain at his peak level, 3 to 6 hours after sciatic nerve block has disappeared. Another consideration arises from the evidence that in both groups the level of pain was mild, and the post-operative pain well controlled through the multimodal post-operative pain management adopted. In fact, VAS values recorded during the study in both groups were sufficiently low to broadly affirm that both our multimodal analgesic modalities are clinically effective. The noticeable number of patients who asked for opioids (more than 70% in both groups) showed that pain after traditional hallux surgery is still a relevant issue and needs to be treated aggressively. Oxycodone is the first choice in ERAS protocols when an opioid is needed for a short period^27^. In our study even if it was allowed as a rescue medication up to 20mg/24h, the average dose in both groups had been approximately 7mg/24h only without any statistical differences between the two groups (p=0.69).

Another point concerns the feasibility of ambulatory surgery with traditional Hallux Valgus procedures. SCARF osteotomy is a well-known traditional procedure that has been popularised by Barouk LS in Europe^17^ and Weil in the Americas^28^. It may be combined with first ray proximal phalanx osteotomies and with lesser metatarsals and lesser toes corrective surgery^17^. SCARF osteotomy correctly conducted should be intrinsically stable, but with screw fixation the patients are immediately capable to bear weight as tolerated, in a standard post-operative shoe^29^. If the patients are then appropriately instructed to bear weight as tolerated and have practiced their level of mobilisation during their hospital stay, they might proceed to a smooth same-day discharge. In our experience, the duration of motor block, evaluated by Bromage Score, had been 3 ± 0.5 hours in each group. We agree that with an appropriate routine together with a multimodal analgesic technique and multimodal pharmacological analgesia prescribed at hospital discharge a significant reduction in stay may be achieved^30^.

## Conclusion

PTNB can represent a valid alternative analgesic technique for post-operative pain control compared to LIA, because it has demonstrated a significantly better control of postoperative pain. Despite VAS assessment both analgesic techniques matched during the study in terms of postoperative motor recovery and analgesic demands. Since VAS values never reached clinically relevant levels in neither of the groups, in a multimodal pain management according to ERAS protocol, both PTNB and LIA should be considered as clinically effective analgesic modalities in traditional hallux valgus surgery. PTNB entails a theoretical risk of nerve injury.
